# Brachial–ankle pulse wave velocity is independently associated with urine albumin-to-creatinine ratio in a Chinese community-based cohort

**DOI:** 10.1007/s11255-020-02404-2

**Published:** 2020-02-25

**Authors:** Yimeng Jiang, Fangfang Fan, Jia Jia, Danmei He, Pengfei Sun, Zhongli Wu, Yong Huo, Yan Zhang

**Affiliations:** Department of Cardiology, Peking University First Hospital, No. 8 Xishiku Street, Xicheng District, Beijing, 100034 China

**Keywords:** Brachial–ankle pulse wave velocity, Urine albumin-to-creatinine ratio, Pathological albuminuria, Threshold-effect analysis

## Abstract

**Purpose:**

Arterial stiffness is important in the development of albuminuria. The brachial–ankle pulse wave velocity (baPWV) acts as an indicator of arterial stiffness and may be associated with cardiovascular disease morbidity and mortality. The urine albumin-to-creatinine ratio (UACR) is a metric used to diagnose albuminuria and has also been shown to be associated with cardiovascular disease. Here, we aim to elucidate the relationship between the baPWV and UACR in the Chinese community.

**Methods:**

A community-based cohort of 3669 subjects was selected for the analysis. The BaPWV and UACR were measured from each subject. UACR ≥ 30 mg/g was defined as pathological albuminuria.

**Results:**

The mean baPWV was 1536.59 ± 305.89 cm/s, and the median UACR value was 6.11 mg/g (interquartile range 4.17, 10.68). A threshold-effect analysis was conducted, and the results showed that the cut-off value for the baPWV was 1269 cm/s. In subjects with baPWV values lower than 1269 cm/s, the prevalence of microalbuminuria and macroalbuminuria was not significantly associated with the baPWV (odds ratio 0.77, 95% confidence interval 0.57–1.03, *P* = 0.08). However, in participants with baPWV ≥ 1269 cm/s, the prevalence of microalbuminuria and macroalbuminuria increased with increasing baPWV 100 cm/s (odds ratio 1.16, 95% confidence interval 1.11–1.22, *P* < 0.001).

**Conclusions:**

These findings suggest that, in this Chinese community-based cohort, elevated baPWV is independently associated with pathological albuminuria with a cut-off value of 1269 cm/s as determined by threshold-effect analysis.

## Introduction

Pathological albuminuria, including macroalbuminuria and microalbuminuria, is used as an early indicator for renal damage, especially in patients with diabetes mellitus and hypertension [1]. Despite its close association with chronic kidney disease, albuminuria has also been shown to predict cardiovascular disease (CVD) morbidity and mortality and all-cause mortality in participants with diabetes and hypertension [2–4]. Furthermore, some studies have indicated that it also holds in the general population [5]. This correlation may be partly attributed to endothelial dysfunction.

The gold standard for measuring urine albumin is 24-h urine collection. However, this method is inconvenient and, therefore, is less widely used than spot urine collection for nephropathy screening. The urine albumin-to-creatinine ratio (UACR) is a commonly used marker for diagnosing albuminuria [6]. A UACR of 30 mg/g is generally regarded as the cut-off value distinguishing normal albuminuria from pathologic albuminuria.

Pulse wave velocity is an index for arterial stiffness and is also independently associated with cardiovascular events. Although carotid-femoral pulse wave velocity (cfPWV) is considered the noninvasive gold standard for measuring arterial stiffness [7], baPWV is widely accepted and used, because it can be measured more simply and quickly, especially in eastern Asia [8]. High baPWV values are associated with increased cardiovascular event morbidity and cardiovascular-related mortality as well as all-cause mortality in patients with diabetes and hypertension and, possibly, the general population [9–12].

Some studies have shown that arterial stiffness is associated with albuminuria in diabetic and hypertensive participants: the higher the baPWV, the higher the albuminuria prevalence [13, 14]. The exact mechanism remains unclear, but investigators have posited that increased arterial stiffness results in glomerular hypertension and that inflammation may cause damage to the glomerular filtration barrier. However, contradictory results have been reported [15]. Therefore, in this cross-sectional study, we aim to investigate the association of baPWV and pathological albuminuria in a Chinese community-based population.

## Methods

### Study population

9540 residents residing in the Gucheng and Pingguoyuan communities of the Shijingshan district in Beijing, China, aged 40 years or older were recruited by calling patients from medical records of community health centers and from people responding to recruitment posters between December 2011 and April 2012 for the initial atherosclerosis cohort. From May to July 2014, 3823 (64.1%) out of 5962 participants with gene chip data who were invited for follow-up responded and attended an onsite visit. Participants without UACR or baPWV measures in 2014 were excluded from the study. Participants with ankle–brachial indexes (ABIs) below 0.9 were also excluded, because it is difficult to measure baPWV precisely in people with peripheral artery disease. Thus, 3669 subjects remained in the analysis. The study protocol was reviewed and approved by the ethics committees of Peking University First Hospital and Peking University, and each subject provided written informed consent. All investigators were trained at the Peking University First Hospital.

### Cardiovascular risk factors, comorbidities, and medications

Demographic and lifestyle information, including age, gender, and smoking and drinking habits, were obtained using standard questionnaires. Current smoking was defined as smoking one cigarette per day for at least 6 months prior. Current drinking was defined as drinking once per week for at least 6 months prior. Medical histories (hypertension, diabetes mellitus, dyslipidemia, and cardiovascular diseases) as well as medication histories (anti-hypertensive agents, anti-diabetic agents, and lipid-lowering agents) were also attained by questionnaire.

Seated brachial BP was assessed three consecutive times in each participant using an Omron HEM-7117 electronic sphygmomanometer with standard calibrations after a 5 min resting period, and the results were averaged. The body mass index (BMI) of each participant was calculated as the subject’s weight (in kg) divided by his or her height squared (in m^2^). Venous blood samples were obtained after 12–15 h of fasting, which separated within 30 min of collection. Each participant’s total cholesterol, low-density lipoprotein (LDL) cholesterol, high-density lipoprotein (HDL) cholesterol, triglyceride, and fasting blood glucose (FBG) concentrations were measured using the Roche C8000 Automatic Analyzer (Beckman Coulter, Inc.). The serum creatinine (Scr; µmol/L) was measured using the same instrument by an enzymatic method. The estimated glomerular filtration rate (eGFR) was estimated based on the Chronic Kidney Disease Epidemiology Collaboration (CKD-EPI) equation as 141 × min(Scr/*κ*, 1)^*α*^ × max(Scr/*κ*, 1)^−1.209^ × 0.993^Age^ × 1.018, where Scr is serum creatinine (mg/dL), *κ* is 0.7 for females and 0.9 for males, *α* is − 0.329 for females and − 0.411 for males, min indicates the minimum of Scr/*κ* or 1, and max indicates the maximum of Scr/*κ* or 1.

Hypertension was defined as systolic blood pressure ≥ 140 mmHg or diastolic blood pressure ≥ 90 mmHg or a self-reported history of hypertension or taking anti-hypertensive agents. Diabetes mellitus was defined as fasting blood glucose level ≥ 7.0 mmol/L or self-reported history of diabetes mellitus, taking anti-glycemic agents, or undergoing insulin therapy. Dyslipidemia was defined as TC ≥ 5.18 mmol/L, TG ≥ 1.70 mmol/L, or LDL cholesterol ≥ 3.37 mmol/L or HDL cholesterol < 1.04 mmol/L or self-reported history of dyslipidemia or taking lipid-lowering agents. The eGFR values’ classification was divided by 90 mL/min/1.73 m^2^ and 60 mL/min/1.73 m^2^. CVD was defined as self-reported history of stroke, transient ischemic attack, or coronary heart disease.

### UACR measurement

Spot morning urine samples were obtained from each participant. Urine albumin was assessed by the bromocresol green method, and urine creatinine was analyzed by the picric acid method using a Unicel DxC 800 Synchron biochemistry analyzer (Beckman Coulter, Inc.) The UACR was defined as the ratio of urine albumin to urine creatinine in units of mg albumin per g creatinine. Normal albuminuria was defined as UACR < 30 mg/g, microalbuminuria was defined as UACR > 30 mg/g and < 300 mg/g, and macroalbuminuria was defined as UACR ≥ 300 mg/g.

### BaPWV measurement

A BP-203RPE III instrument (Colin-Omron, Co. Ltd) was used to measure the baPWV from each subject. After the participant rested in the supine position for at least 5 min, a trained investigator wrapped cuffs around both arms and legs and recorded the pulse waveforms from the cuffs simultaneously. The baPWVs were automatically calculated by the instrument, and the higher one of the two bilateral baPWVs was recorded for subsequent analyses.

### Statistical analysis

Normally distributed continuous variables were expressed as mean ± standard deviation (SD). For skewed distributed variables, the median of the interquartile range was used. Categorical variables were reported as *n* (%). Normally distributed variables were compared using a t test, skewed distributed variables were compared using a Kruskal–Wallis test, and categorical variables were compared using the Pearson Chi-square test or Fisher’s exact test. Multivariate logistic regression models were used to investigate the association between baPWV and pathological albuminuria after adjusting for age, gender, current smoking and drinking habits, BMI, hypertension, diabetes mellitus, dyslipidemia, anti-hypertensive agents, anti-diabetic agents, lipid-lowering agents, and self-reported history of CVD. A threshold-effect analysis was also performed. All statistical analyses were performed using Empower(R) (www.empowerstats.com, X&Y solutions, Inc., Boston, MA, USA) and R software (www.R-project.org). A two-sided *P *< 0.05 was considered significantly significant.

## Results

### Baseline characteristics

The baseline characteristics and albuminuria classifications of all study participants are shown in Table 1. The participants were 58.86 ± 8.44 years old on average and 35.87% were males. The average BMI was 25.94 ± 3.48 kg/m^2^. 15.66% of the participants were current smokers and 14.11% of them were current drinkers. Among the participants, 12.27% of the participants had self-reported histories of CVD, 45.71% had hypertension, 19.84% had diabetes mellitus, and 75.69% had dyslipidemia. 25.70% were taking anti-hypertensive agents, 9.08% were taking anti-diabetic agents, and 9.62% were taking lipid-lowering agents. According to the criteria, 8.15% of the participants had eGFRs > 90 mL/min/1.73 m^2^, 82.93% had an eGFRs in the range of 90 mL/min/1.73 m^2^ to 60 mL/min/1.73 m^2^, and 8.92% had eGFRs<60 mL/min/1.73 m^2^.

**Table 1:** Baseline characteristics of eligible participants, including their UACR classifications

	Total	UACR < 30 mg/g	UACR ≥ 30 mg/g	*P* value
Age (years)	58.86 ± 8.44	58.64 ± 8.22	61.62 ± 10.35	< 0.001
Gender (male)	1316 (35.87%)	1197 (35.31%)	119 (42.65%)	0.014
BMI (kg/m^2^)	25.94 ± 3.48	25.89 ± 3.46	26.62 ± 3.61	< 0.001
Current smoking	573(15.66%)	523 (15.47%)	50 (17.99%)	0.268
Current drinking	516 (14.11%)	458 (13.55%)	58 (20.86%)	< 0.001
eGFR classification				< 0.001
≥ 90 mL/min/1.73 m^2^	298 (8.15%)	278 (8.23%)	20 (7.17%)	
60-90 mL/min/1.73 m^2^	3032 (82.93%)	2830 (83.80%)	202 (72.40%)	
< 60 mL/min/1.73 m^2^	326 (8.92%)	269 (7.97%)	57 (20.43%)	
Max PWV	1536.59 ± 305.89	1520.82 ± 289.82	1728.24 ± 413.20	< 0.001
UACR (median, IQR)	6.11 (4.17, 10.68)	5.75 (4.03, 9.14)	63.03 (39.07, 131.34)	< 0.001
Hypertension	1677 (45.71%)	1481 (43.69%)	196 (70.25%)	< 0.001
Diabetes mellitus	726 (19.84%)	623 (18.43%)	103 (37.05%)	< 0.001
Dyslipidemia	2777 (75.69%)	2548 (75.16%)	229 (82.08%)	0.010
Anti-hypertensive agent	940 (25.70%)	831 (24.59%)	109 (39.21%)	< 0.001
Anti-diabetic agent	332 (9.08%)	291 (8.61%)	41 (14.75%)	< 0.001
Lipid-lowering agent	352 (9.62%)	323 (9.56%)	29 (10.43%)	0.634
CVD	449 (12.27%)	393 (11.63%)	56 (20.14%)	< 0.001

### Major indexes

The median UACR value was 6.11 mg/g (IQR 4.17–10.68 mg/g). According to the definition of albuminuria, 92.40% of the participants had normal albuminuria, 6.76% had microalbuminuria, and 0.84% had macroalbuminuria. The average baPWV was 1536.59 ± 305.89 cm/s. Participants with microalbuminuria and macroalbuminuria were older and had higher BMIs, were more likely to be males and current drinkers, and more often had histories of hypertension, diabetes mellitus, dyslipidemia, and CVD.

**Fig. 1:**
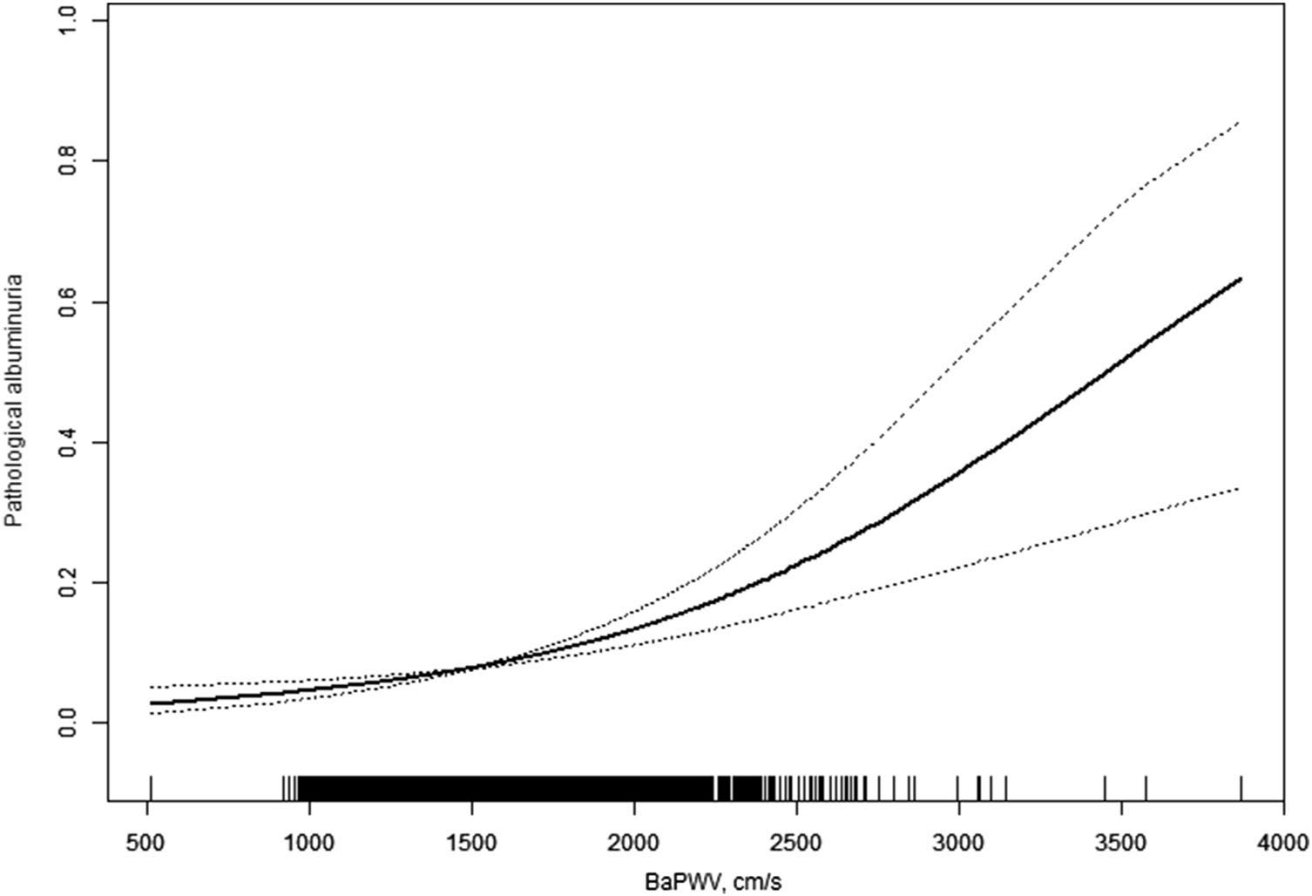
Association between baPWV value and  pathological albuminuria

Two models were used to evaluate the relationship between baPWV and pathological albuminuria. In the logistic regression model, baPWV was associated with pathological albuminuria [odds ratio (OR) = 1.14, 95% confidence interval (CI) 1.09–1.20, *P* < 0.001] with every 100 cm/s increase in baPWV (Fig. 1).

Given the non-linear relationship between baPWV and pathological albuminuria, a threshold-effect analysis was also conducted. The threshold analysis was adjusted for gender, age, BMI, smoking and drinking status, eGFR classification, and histories of hypertension, diabetes mellitus, dyslipidemia, CVD, anti-hypertensive agents, anti-diabetic agents, and lipid-lowering agents (Table 2). The cut-off value for baPWV was found to be 1269 cm/s. Results showed that in subjects with baPWVs lower than 1269 cm/s, the presence of microalbuminuria and macroalbuminuria was not significantly associated with baPWV (OR 0.77, 95% CI 0.57–1.03, *P* = 0.008). However, in participants with baPWV of 1269 cm/s or higher, the rate of microalbuminuria and macroalbuminuria increased with the baPWV increasing 100 cm/s (OR 1.16, 95% CI 1.11–1.22, *P* < 0.001). Furthermore, a log-likelihood ratio test was performed, and the results indicated statistically significant differences between two slopes below and above a baPWV of 1269 cm/s (*P* = 0.017). An interaction test was performed with participants with baPWVs ≥ 1269 cm/s using the modification factors mentioned above, as shown in Table 3. The results showed that there was no statistically significant interaction.

**Table 2:** Threshold-effect analysis of the relationship between baPWV and pathological albuminuria using a piecewise linear regression model

Model	OR (95% CI)	*P* value
Model I: one-line	1.14 (1.09, 1.20)	< 0.001
Model II: turning point: 1269 cm/s		
Slope 1: baPWV< 1269 cm/s	0.77 (0.57, 1.03)	0.080
Slope 2: baPWV ≥ 1269 cm/s	1.16 (1.11, 1.22)	< 0.001
Slope 2-Slope1	1.51 (1.11, 2.05)	0.008
A log-likelihood ratio	0.017	

The odds ratios (ORs) represent the effects for every 100 cm/s increase in baPWV

Adjusted for sex, age, BMI, smoking status, drinking status, eGFR classification, hypertension, diabetes mellitus, dyslipidemia, use of anti-hypertensive, anti-diabetic, and lipid-lowering agents, and CVD history

**Table 3:** Multivariate-adjusted subgroup odds ratios (ORs) for every 100 cm/s increase in baPWV among subjects with UACR ≥ 30 mg/g compared with those having UACR< 30 mg/g

Variable	Subjects (*n*)	Multivariable-adjusted models	
		OR (95% CI)	*P* interaction
Gender			
Male	1222	1.19 (1.11, 1.26)	0.146
Female	1921	1.12 (1.06, 1.18)	
Age			
< 60 years	1676	1.14 (1.05, 1.24)	0.943
≥ 60 years	1467	1.15 (1.09, 1.21)	
BMI			
< 28 kg/m^2^	2304	1.13 (1.08, 1.19)	0.380
≥ 28 kg/m^2^	839	1.18 (1.09, 1.27)	
Current smoking			
No	2630	1.14 (1.08, 1.19)	0.307
Yes	513	1.21 (1.08, 1.34)	
Current drinking			
No	2680	1.14 (1.08, 1.19)	0.443
Yes	463	1.19 (1.07, 1.31)	
eGFR			
< 60 mL/min/1.73 m^2^	335	1.17 (1.08, 1.27)	0.471
≥ 60 mL/min/1.73 m^2^	2808	1.14 (1.08, 1.19)	
CVD			
No	2619	1.15 (1.09, 1.21)	0.651
Yes	524	1.13 (1.05, 1.22)	
Hypertension			
No	1491	1.17 (1.07, 1.28)	0.619
Yes	1652	1.14 (1.09, 1.20)	
Diabetes mellitus			
No	2414	1.16 (1.10, 1.22)	0.510
Yes	729	1.13 (1.06, 1.20)	
Dyslipidemia			
No	709	1.15 (1.04, 1.26)	0.966
Yes	2434	1.14 (1.09, 1.20)	
Anti-hypertensive agent			
No	2227	1.17 (1.11, 1.24)	0.148
Yes	916	1.11 (1.04, 1.18)	
Anti-diabetic agent			
No	2809	1.16 (1.11, 1.22)	0.183
Yes	334	1.09 (1.00, 1.19)	
Anti-dyslipidemia agent			
No	2819	1.14 (1.09, 1.20)	0.850
Yes	324	1.16 (1.01, 1.33)	

Multivariate models adjusted for sex, age, BMI, smoking status, drinking status, eGFR classification, hypertension, diabetes mellitus, dyslipidemia, use of anti-hypertensive, anti-diabetic agent, and lipid-lowering agents, and CVD history

## Discussion

In our cross-sectional study, we found that baPWV was associated with pathological albuminuria in a Chinese community-based cohort. A threshold-effect analysis revealed that the cut-off point for the baPWV was 1269 cm/s. When baPWV was lower than 1269 cm/s, baPWV was not associated with pathological albuminuria. However, when baPWV was higher than 1269 cm/s, the risk of having microalbuminuria and macroalbuminuria increased as the baPWV increased. To our knowledge, this is the first study validating an association between baPWV and pathological albuminuria with a threshold.

Microalbuminuria and macroalbuminuria are markers for target renal damage in diabetic and hypertensive patients. It occurs via a mechanism involving increased vascular permeability related to endothelial damage. As the morbidity of chronic kidney disease is increasing worldwide [16], which imposes a heavy burden on the social system, the early detection of CKD from albuminuria is critical as it could permit early intervention [17] and prevent cardiovascular complications.

The prevalence of pathological albuminuria varies across regions and populations. In the general Chinese population, the prevalence of pathological urine albumin was 9.4% [18]. However, among hypertensive type 2 diabetic patients in mainland China, the prevalence of microalbuminuria was 42.9% and that of macroalbuminuria was 17.0% [19]. In the United States, the microalbuminuria prevalence has been reported as 8.2%, and the macroalbuminuria prevalence has been reported as 1.3% [20]. The prevalence of pathological albuminuria in the general population in Japan has been found to be 14.9% [21]. In the community-based Chinese cohort in the present study, the prevalence of microalbuminuria was 6.76%, that of macroalbuminuria was 0.84%, and the total prevalence of abnormal albuminuria was 7.60%. In a community-based cross-sectional study of people of Han ethnicity aged 60–95 years in Beijing, China, the overall rate of pathological albuminuria was 13.16%; this was higher than the 8.25% reported in our study, which can be attributed to the relatively old age of the study population [22]. In another study of a rural population aged over 40 years in Korea, the prevalence of microalbuminuria was 9.9% [23].

BaPWV is a metric to estimate arterial stiffness and reflects the stiffness of the aorta and peripheral arteries. It is more readily applicable in the clinic than cfPWV, since it is automated and easier to measure. The mean baPWV in our study was 1536.59 ± 305.89 cm/s, which is lower than those in Xu’s study, which reported an average baPWV of 2154.50 ± 502.97 cm/s in the populations with an ABI < 0.9 and 2049.49 ± 456.65 cm/s in populations with ABI ≥ 0.9 [22]. The difference between baPWV values may be partly due to the old age of the study participants in the study by Xu (76.17 ± 6.05 years in the ABI < 0.9 group and 71.41 ± 6.48 years in the ABI ≥ 0.9 group), because age is one of the major determinants of PWV. In addition, the prevalences of hypertension (65.8% in the ABI < 0.9 group and 54.1% in the ABI ≥ 0.9 group) and diabetes mellitus (28.9% in the ABI < 0.9 group and 18.3% in the ABI > 0.9 group) were also higher than in our study, which may also contribute to stiffer arteries. In a study conducted in Korean rural communities with participants aged over 40 years, the median baPWV was 1515 cm/s (interquartile range 1325–1764 cm/s), which is similar to that in the present study [23].

Our findings were consistent with those of another community-based cross-sectional study of 2127 community residents of Han ethnicity in Beijing, China aged 60–95 years [22]. The participants were divided into quartiles according to baPWV value: < 1730, 1730–1983, 1983–2305, and > 2305 cm/s. They found that baPWV was significantly associated with albuminuria in this cohort; the ORs of having pathological albuminuria in baPWV quartiles II, III, and IV were 1.07 (OR, 95% CI 0.67, 1.69), 1.15 (OR, 95% CI 1.07, 1.32), and 1.23 (OR, 95% CI 1.04, 1.46), respectively. The baPWV cut-off value was much higher than that in our study. Although both studies were conducted with Chinese community-based cohorts, the previous study was conducted with an elderly population and excluded residents with histories of CVD and stroke, both of which may contribute to the higher observed baPWV. In a previous Korean study of a rural population of 1648 participants aged over 40 years [23], the baPWV was proven to be independently associated with microalbuminuria; after adjusting for several variables, log(baPWV) was still an independent risk factor for microalbuminuria in both men (OR 15.813, 95% CI 2.629–95.119) and women (OR 5.399, 95% CI 1.157–25.205). Unlike our study, this study excluded participants with CVD and stroke and those with macroalbuminuria and overt albuminuria. Liu et al. found that albuminuria was strongly associated with baPWV, especially in diabetic or hypertensive patients. Subjects were divided into quartiles based on their baPWVs: < 1368, 1368–1572, 1573–1857, and > 1858 cm/s in men and < 1256, 1256–1444, 1445–1752, and > 1753 cm/s in women. Compared with the lowest baPWV quartile, the adjusted ORs of having albuminuria for baPWV quartiles II, III, and IV were 1.12 (OR, 95% CI 0.63, 2.02), 2.04 (OR, 95% CI 1.15, 3.60), and 2.45 (OR, 95% CI 1.29, 4.65), respectively. The significant increase in OR for albuminuria between quartiles with increasing baPWVs was thought to reflect a dose–response effect (*P* < 0.001). Moreover, these relationships were stronger among patients with diabetes, hypertension, and macroalbuminuria compared with those without these conditions [13]. In this study, we performed an interaction test and found that there was no interaction effect between hypertension and diabetes mellitus. This finding not only confirms the association between baPWV and microalbuminuria in diabetic and hypertensive patients, but also provides supporting evidence that the relationship holds in the broader community. Furthermore, the J-Topp study showed that a high baPWV was a predictor of new-onset microalbuminuria in a non-diabetic population of 321 participants with essential hypertension in a 2 year follow-up [24]. However, to our knowledge, there is no predictive study concerning baPWV and UACR in a general community-based population.

Besides, Matsui et al. found that in hypertensive patients, the arterial stiffness reduction measured by baPWV was associated with the improvement of renal damage evaluated by UACR change, and the effect was independent of home and office blood pressure reduction. Therefore, reducing arterial stiffness may be an important strategy for preventions of albuminuria progression and cardiovascular events [25]. Therefore, our result may provide further evidence for the association between baPWV reduction and UACR improvement, but still, further prospective studies were needed to validate it.

Generally, a baPWV of 1400 cm/s is deemed as the cut-off value to screen patients, especially middle-aged patients, including both males and females [26]. Our findings provide evidence for a baPWV cut-off value of 1269 cm/s, which is lower than the cut-off value 1400 cm/s that is generally used clinically to diagnose arterial stiffness. This difference may be because the 1400 cm/s cut-off was selected mainly based on outcomes related to large vessels. However, as albuminuria is mainly associated with the microvasculature, pathological albuminuria may not occur, while the arterial stiffness is within the normal range. However, as the baPWV increases, the arterial stiffness may contribute to pathological albuminuria. Based on our findings, when patients present with baPWVs higher than 1269 cm/s, clinicians should be aware of their elevated risk of having pathological albuminuria.

The mechanism of the relationship between baPWV and UACR remains unclear. Albuminuria is considered an early marker of systemic microvascular damage. Some researchers believe that baPWV is an indicator of arterial stiffness, which, in turn, contributes to endothelial dysfunction and increases the permeability of the glomerular basement membrane, leading to albuminuria [27]. Furthermore, higher baPWVs cause glomerular damage via increased pulsatile stress because of the luxury flow and low resistance in the kidney [28]. Inflammation may also play a role in the atherosclerotic process and kidney injury [29].

## Strength and limitations

Our study was the first study to demonstrate conclusively that baPWV has a threshold effect with pathological albuminuria after adjusting for risk factors in a community-based Chinese cohort. Our findings revealed a cut-off value for baPWV that may be used to screen patients at high risk of developing pathological albuminuria and may provide a specific target for drug therapy, such as indicating whether renin–angiotensin–aldosterone system (RAAS) inhibitors should be prioritized. However, there were several limitations in our study. The main limitation was it was a cross-sectional study, and further follow-up is required to validate the cause–effect association. Second, we did not ask participants with hypertension if they were taking RAAS inhibitors. However, after adjusting for the use of anti-hypertensive agents, the relationship between baPWV and pathological albuminuria remains. Another limitation is that baPWV was used instead of cfPWV, which is the gold standard. However, as several studies have demonstrated that baPWV and cfPWV are equally related to cardiovascular risk factors, we chose baPWV as a substitute [30, 31]. In addition, urinary albumin excretion was measured from single-spot urine samples rather than 24-h urine samples in our study. Nevertheless, UACR has been proven to be highly correlated with 24-h urine albumin excretion [32] and has been widely applied in other studies. Finally, it is indeed a post hoc analysis which is used for hypothesis-generating, and studies were warranted to further prove the finding of our research.

## Conclusions

Based on our findings, we concluded that increased baPWV is independently associated with pathological albuminuria in a Chinese community-based cohort. A threshold-effect analysis revealed a baPWV cut-off of 1269 cm/s, above which patients have elevated risk of microalbuminuria and macroalbuminuria. This finding supports the theory that arterial stiffness plays an important role in the formation of albuminuria. These results should raise awareness among clinicians regarding the elevated risk of albuminuria in patients with high baPWVs.
